# Climat change and mental health: an urgent challenge :the heat is on

**DOI:** 10.1192/j.eurpsy.2026.10991

**Published:** 2026-07-31

**Authors:** Z. Souhlal

**Affiliations:** Hospital, Vernon, France

## Abstract

**Introduction:**

A significant rise in global temperatures has become evident in recent years. Climate change and global warming are now established realities, not future projections. The impact on human health is increasingly documented, yet the mental health dimension remains underexplored. Anxiety disorders, climate-induced clinical conditions, and the worsening of pre-existing psychiatric disorders constitute urgent challenges. Such consequences may also foster the emergence of new psychopathological disorders.

**Objectives:**

Early identification of climate- related menta health issues. Preventive interventions to reduce the risk of anxiety,mood and stress related disorders. Comprehensive care approaches integrating psychological support, community resources, and resilience building programs. Training for mental health professionals to recognize and manage emerging climate related psychopathologies.

**Methods:**

Various reports highlight the potential increase in post traumatic stress disorders (PTSD) due to the intensification of natural disasters and climate related migration. IPCC (Intergovernmental Panel on Climate Change) reports point to the growing mental health burden associated with climate change. Findings from the NIH (National Institutes of Health) also underscore the psychological consequences of environmental disruptions

**Results:**

Eco-anxiety and solastalgia must be identified and adressed to prevent the onset of new anxiety disorders and potential mood disorder decompensations. The seasonal compenent of bipolar disorder may also be significantly affected to further contribute to mental health disorders. The impact of climat change on addictive behaviors and suicidal tendencies must also be taken into account Early detection, prevention and appropriate management of these conditions should be considered a priority for all mental health professionals

**Conclusions:**

There is an urgent need to conduct and expand studies demonstrating the impact of climate change on human mental health. Climate change represents a **risk and vulnerability factor** for the deterioration of mental health. **Climate action** itself can serve as both an intervention and a means of mitigating mental health decline.

**Disclosure of Interest:**

None Declared

